# Assessment of Leaching Characteristics of Solidified Products Containing Secondary Alkaline Lead Slag

**DOI:** 10.3390/ijerph16112005

**Published:** 2019-06-05

**Authors:** Marija Štulović, Dragana Radovanović, Željko Kamberović, Marija Korać, Zoran Anđić

**Affiliations:** 1 Innovation Center of Faculty of Technology and Metallurgy in Belgrade Ltd., University of Belgrade, Karnegijeva 4, 11000 Belgrade, Serbia; divsic@tmf.bg.ac.rs; 2 Faculty of Technology and Metallurgy, University of Belgrade, Karnegijeva 4, 11000 Belgrade, Serbia; kamber@tmf.bg.ac.rs (Ž.K.); marijakorac@tmf.bg.ac.rs (M.K.); 3 Innovation Center of Faculty of Chemistry in Belgrade Ltd., University of Belgrade, Studentski trg 12–16, 11000 Belgrade, Serbia; zoranandjic@chem.bg.ac.rs

**Keywords:** slag, lead, arsenic, leaching, solidification/stabilization, geochemical modeling

## Abstract

Reuse of waste is one of the main principles of sustainable development and circular economy. Secondary alkaline lead slag is a hazardous waste generated in the recycling process of lead-acid batteries that may be suitable in construction materials. The environmental impact of the use of lead slag as a partial replacement of fine aggregates in the cement-based stabilization/solidification (S/S) process for the preparation of concrete was studied in this paper. Solidified products containing 10%, 15%, 20%, and 25% slag were laboratory tested by unconfined compressive strength (UCS) analyses and the Toxicity Characteristic Leaching Procedure (TCLP). At the same time, the leachability of toxic elements from solidified products with a high percent of slag was evaluated under environmental conditions for during one year. The results of the UCS and TCLP indicated that utilization of this type of slag in cement-based applications may be justified with its controlled addition. However, the described application of the slag was disputed due to the high release of As under high alkaline environmental conditions. Eh-pH analyses and the geochemical modeling using the software PHREEQC were evaluated, as well as the mechanism of pollutant (Pb, As) immobilization (precipitation, adsorption) as a function of pH conditions.

## 1. Introduction

Lead is a strategically important metal for industrial development with significant applications within the global economy, and with well-established patterns of primary and secondary production. Globally, the demand for refined lead metal in the year 2018 was 11.90 million tons and it is set to peak within 15 years with predictions that the demand for lead for battery production will increase to 14 Mt/y in 2025 [1,2]. The manufacturing of batteries, the most economical and effective system to store energy [3], is the largest consumption sector of lead metal. At the same time, used lead-acid batteries are the main resource of secondary lead production, forming 85% of the total amount of secondary lead [4].

Most of the secondary lead recovery plants are based on pyrometallurgical methods, which generate lead slag (solid waste) in an amount of 13–25% of produced lead [5,6]. Apart from lead compounds (approximately 5%), these slags contain migratory toxic elements that pollute the environment under weathering and leaching conditions [7]. The leachability of toxic elements from lead slag constitutes a serious impact on the environment and human lives [8]. The potential release of toxic elements is influenced by the used fluxes, the operating conditions, and by mineral phases formed in the slag [6]. The use of sodium-based fluxes (Na_2_CO_3_) to fix sulfur causes an increase in the pollution potential of lead slag due to its high leachability, hygroscopic, and pulverized nature with a self-igniting effect. Nature of this type of slag was investigated by previous authors’ research [8,9]. They pointed to the reaction of Na_2_S from the slag with water and CO_2_ from the air, which leads to the formation of Na_2_CO_3_·H_2_O. This reaction was followed by an increase of total weight and finer fraction (1 mm) portion of the slag from 5 to 85% during a storage period of 30 days. Iron compound FeS_2_ affects self-ignition behavior of coke remaining in the slag [10]. Due to the high pollution potential of the generated slag, new regulations are requiring more environmentally friendly technologies for the lead production industry [6,11].

The management of lead slag is based on the prevention of the negative impact on the environment (decreasing toxicity) and favors a circular economy (increasing utilization) [5]. The S/S process is used for the treatment of lead slag with the toxic elements immobilization (physical encapsulation, adsorption, and ion exchange) in the geopolymer matrix [12] and with physical encapsulation and chemical fixation (dissolved into a crystal structure) in alkali-activated cementitious material [13]. The preferred technology to treat hazardous waste is metal recovery and reuse of the slag in construction materials [5]. The slag from the battery recycling process may be suitable for construction use depending on the leachability of the metals they contain [6]. Extremely high leaching of Ba under long-term leaching time (12 years) limited the utilization of lead slag as construction materials according to the study of Ettler et al. [14]. However, Saikia et al. [15] confirmed the indication that the lead slag from primary lead production could be used as a partial replacement of fine aggregates in cement mortars and concrete without negative effects of the products of the slag hydration with cement to the properties of the cement materials. The other study of Saikia et al. [16] pointed that the use of slag as a raw material in cement-based product could cause environmental problems due to the potential release of toxic elements into the environment. The release of toxic elements was controlled by the pH of the leaching solution and the leaching tests are the basic tests which create the criteria for further utilization of industrial waste [17]. A technical and environmental evaluation of this concept was performed in previous research studies [9,18]. Results of the TCLP and NEN 7375 tests showed that secondary lead slag could be used as a component in concrete production, but only with careful control of the solidification process and in the presence of selected additives (magnesium oxide, calcium oxide, barium hydroxide, and gypsum).

The geochemical PHREEQC software [19] was used to model the leaching processes of toxic elements from solidified products of industrial wastes and construction materials. PHREEQC is a computer program that is designed to perform a wide variety of aqueous geochemical calculations. PHREEQC is based on an ion-association aqueous model and has capabilities for (1) speciation and saturation-index calculations, (2) reaction-path and advective-transport calculations involving specified irreversible reactions, mixing of solutions, mineral and gas equilibria, surface-complexation reactions, and ion-exchange reactions, and (3) inverse modeling, which finds sets of mineral and gas mole transfers that account for composition differences between waters, within specified compositional uncertainties. The program is based on the coupling of the geochemical model with transport phenomena, allowing the simulation of the slow passage of liquid through a filtering medium or its diffusion in a simple system.

The aim of this work was the assessment of the leaching behavior of toxic elements (Pb and As) from solidified products containing the slag under both standard laboratory and real environmental conditions. An industrially produced secondary alkaline lead slag was incorporated into the solidified products as partial replacements of fine aggregates. Mechanical and leaching characteristics of solidified products containing the slag (10%, 15%, 20%, and 25%) were determined by the standard UCS procedure and TCLP test. In order to investigate the actual leaching behavior of contaminants from solidified products in a real environment, the cement-based solidified products were exposed to atmospheric conditions for one year. The results of standard leaching test and leaching under environmental conditions were compared. The results of the simulation obtained by the geochemical modeling using the PHREEQC program [19] were analyzed to define the component immobilization mechanism by sorption, precipitation, or the combination of interaction, in a multi-component system. The results provide further explanation of the limitations in the reuse of this type of hazardous waste.

## 2. Materials and Methods

### 2.1. Materials 

The fine fraction (0–1 mm) of alkaline slag, generated in the recycling process of waste lead-acid batteries with soda ash as the fluxing agent, was sieved after a storage period of 30 days and used as a partial replacement of fine aggregates in cement-based stabilization/solidification (S/S) processes for the preparation of concrete. Solidified products were prepared by mixing Portland cement (PC 35 M (V-I) 32.5 R), fine aggregate (limestone origin, 0–2 mm) with the slag and coarse aggregate (volcanic origin, 2–4 mm), in the corresponding proportions (Table 1), so that they contained 10%, 15%, 20%, and 25% of the slag by weight. Contents of PC (30%) and coarse aggregate (30%) were constant in all samples.

All samples were made with constant water to binder ratio (w/b) of 0.63 by weight, where the required workability was met. Mixtures were cast into molds (15 × 15 × 15 cm) and vibrated in order to remove entrapped air and excess water. Samples were cured under wet towels during the first 24 h. After three days, the samples were removed from the molds and left to cure in air.

### 2.2. Characterization of Materials

The chemical compositions of the slag and solidified products (Table 2) were determined by acid digestion. 

A mass of 0.2 g of slag and solidified sample were dissolved in a mixture of concentrated mineral acid containing 9 mL of HNO_3_ and 3 mL of HF for 15 min using microwave heating at 180 ± 5 °C [20]. After cooling, the solution was filtered (Millipore 0.45 μm). The concentrations of the elements were determined by Inductively Coupled Plasma-Optical Emission Spectrometry (ICP-OES, Agilent Technologies, Santa Clara, CA, USA). The analyses were run in triplicate. The content of total sulfur (TS) and total inorganic carbon (TIC) in all samples was determined by an elemental analyser Vario EL III CHNS + O (Elementar Analysensysteme GmbH, Langenselbold, Germany).

### 2.3. The Unconfined Compressive Strength (UCS)

The effectiveness of the stabilization/solidification (S/S) process was assessed by determining the unconfined compressive strength (UCS) of solidified products after 28 days of curing. UCS was measured using a servohydraulic testing machine type INSTRON 1332-retrofitted Fast track 8800 with a maximum load of 100 kN. Results were the mean measurement values of the three samples.

### 2.4. Leaching Behavior of Solidified Products 

#### 2.4.1. Toxicity Characteristic Leaching Procedure 

The pollution potential from the leaching of toxic elements from solidified products was examined by the TCLP test [21]. The procedure was performed at an L/S ratio of 20 glacial acetic acid solution (pH 2.88 ± 0.05), by rotating the mixtures in bottles for 18 h. Solidified products were grounded to pieces of up to 10 mm in size and then subjected to the TCLP test.

#### 2.4.2. Leaching under Environmental Conditions 

The solidified mixture (S25) with the highest amount of slag was chosen for analyses of the solidified product leachability under environmental conditions (LEC), atmospheric water, drying and wetting, and temperature changes for one year. The sample was placed in a plastic bin connected to a plastic tank for the collection of drainage water. A ratio of L/S 1 was determined in accordance with the average annual amount of atmospheric precipitation in Belgrade municipality and the mass of the solidified sample. A sampling of drainage water was planned after six and 12 mounts. 

### 2.5. Analytical Procedures 

The solutions after leaching tests were filtered (Millipore 0.45 μm) and acidified to pH 2 with HNO_3_ p.a. prior to the determination of selected elements concentration by Inductively Coupled Plasma Mass Spectrometer (ICP-MS, Skyray Instrument, Stoughton, WI, USA). Triplicate assays were carried out. The redox potential (Eh) and pH values of solutions were measured using an InoLab 720 pH-meter (Gemini BV, Apeldoorn, Nederland). 

## 3. Geochemical Modeling (PHREEQC Program) 

The PHREEQC program [19] was used to simulate the concentrations of Pb and As leached from solidified products and pH values of leachates during the time. The simulation was based on the calculation of the equilibrium between the aqueous solutions and the minerals, solid solutions, exchangers, and sorption surfaces. The program results were the speciation and concentrations of the elements, as well as the degree of saturation of the leachate with respect to mineral phases as a function of leaching time. 

Thermodynamic data were obtained using the HSC Chemistry 6.12 software [22] for solubility products and dissolution reactions of mineral phases. The thermodynamic data of CaH_2_SiO_4_ (CSH gel) employed were taken from the study of Cheryle et al. [23].

### Model Input

In the model simulation with the PHREEQC geochemical package, the matrix of the solidified product was classified into calcite (CaCO_3_), portlandite, (Ca(OH)_2_), and CSH matrix that was assumed to contain CaH_2_SiO_4_ (CSH gel); Ca_3_Al_2_(H_4_O_4_)_3_; Ca_4_Al_2_Fe_2_O_10_; NaOH; KOH; Mg(OH)_2_ CaSO_4_·2H_2_O, and compounds of Pb(OH)_2_ and Ca_3_(AsO_4_)_2_ [21]. Gel CSH (CaH_2_SiO_4_), Ca_3_Al_2_(H_4_O_4_)_3_, and Ca_4_Al_2_Fe_2_O_10_ represent the solubility-controlling phases and Pb and As were predominantly dispersed throughout the C–S–H matrix [24,25]. 

The composition of the classified matrix in the solidified product was calculated based on the amounts of the elements (S25, Table 2). The amount of CaH_2_SiO_4_ was calculated according to the Si content, the amount of calcite based on the C content, the amount of gypsum on the S content, and the amount of Ca_4_Al_2_Fe_2_O_10_ according to the Fe content. The amount of Ca_3_Al_2_(H_4_O_4_)_3_ was calculated by deduction of the Al content in the Ca_4_Al_2_Fe_2_O_10_ from the total Al content. The Ca(OH)_2_ content was calculated by deduction of the amounts of Ca in the CaH_2_SiO_4_, CaCO_3_, CaSO_4_·2H_2_O, Ca_3_Al_2_(H_4_O_4_)_3_; Ca_4_Al_2_Fe_2_O_10_ from the total Ca content.

A simulation was performed for an L/S ratio of 1 that was complied with the L/S ratio of the leaching experiment under environmental conditions. Distill water (pH 7.0) was defined to be the leaching fluids in this simulation, and it was assumed that 25% of the matrix was available for leaching due to the neutral, non-aggressive nature of leaching fluid, in accordance with the study of Cheryl et al. [23]. 

The initial mass of minerals used to represent the leaching behavior of toxic elements from the solidified product with the corresponding thermodynamic data of reactions (log solubility const. (Ksp), log neutralization const. (Kn), the standard Gibb’s free energy change (ΔG^0^), the standard enthalpy change (ΔH^0^), and the standard entropy change (ΔS^0^)), in the standard state (1 atm of pressure and 298.15 K) [22], are shown in Table 3. 

The amount of metal ions released from the solidified product is determined by the dissolution kinetics of minerals contained in the matrix of the solidified product over any defined leaching period. The dissolution rates of the matrices in the solidified product were obtained from the calcite dissolution rate [26] and they are described in Equation (1).

(1) $$\frac{dM}{dt}=\frac{A}{V}\cdot \left(c_{1}\cdot a_{H+}+c_{2}\cdot a_{H2CO3}+c_{3}\;\cdot a_{OH-}-c_{4}\cdot a_{Ca+2\;}\cdot a_{CO-3}+c_{5}\right)$$

where $\frac{dM}{dt}\;$ is the dissolution rate of the matrix (CaCO_3_, Ca(OH)_2_, CSH matrix); *V* is the volume of the solution per gram of waste; *A* is the surface area of the matrix; $a_{H+}$, $a_{H2CO3}$, $a_{OH-}$, $a_{Ca+2}$, and $a_{CO3-2}$ are the activities of H^+^, H_2_CO_3_, OH^−,^ Ca^+2^, and CO_3_^−2^, respectively. The constants of each component of the solidified product matrix [23] are sown in Table 4. 

Simulation by the PHREEQC program determines the metal speciation by the establishment of thermodynamic equilibrium between the aqueous solutions and minerals, solid solutions, exchangers, and sorption surfaces.

The adsorption phenomena with the surface complexation model of Pb and As on hydrous ferric oxides (Hfo) and silica gel (Surf) [27,28,29,30] were considered. Thermodynamic data (log formation constant (Kf) and the dissociation constant (Kd)) [27,28,29] for adsorption with the surface complexation phenomenon of the metal ion used in the program are shown in Table 5.

Saturation indices (SI) are the ratio of the logarithm of the ion activity product to the logarithm of the solubility product. A positive SI indicates precipitation of the mineral, while a negative SI indicates mineral dissolution. Phases with SI between −2.0 and 2.0 are most likely to be the controlling mineral for constituent element solubility with first precipitation of the minerals (in similar forms, e.g., oxides, hydroxides, carbonates) with the smallest positive SI [23].

## 4. Results

### 4.1. Influence of Slag Contents on Compressive Strength

**Figure 1:**
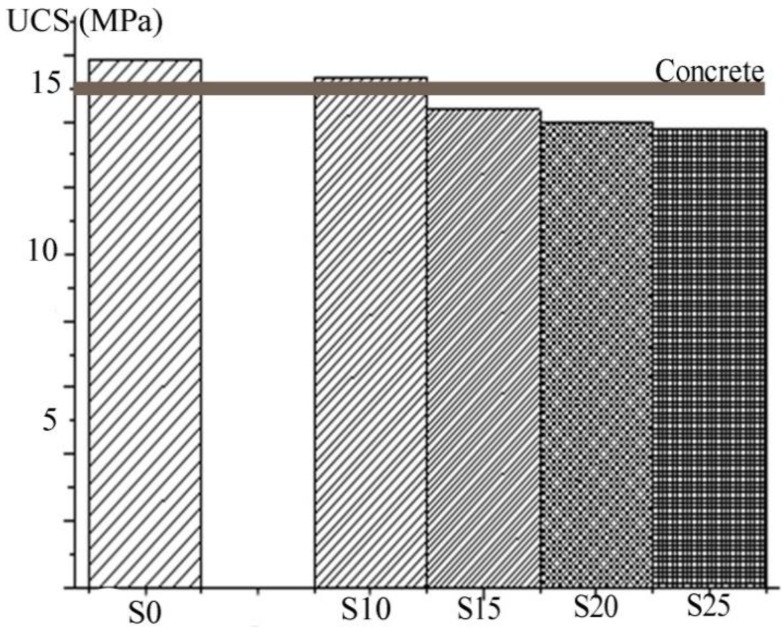
The unconfined compressive strengths of the solidified products S0, S10, S15, S20, S25.

The results of the UCS measurements of solidified products (S0, S10, S15, S20, S25) after 28 days of curing are shown in Figure 1. 

The highest UCS (15.32 MPa) (Figure 1) was achieved in the solidified product with minimal slag content (S10). This value corresponded to the UCS (15.87 MPa) of the solidified product prepared without slag (S0). It was observed that mechanical properties were slowly decreasing with increasing content of slag in solidified products. The lowest UCS (13.75 MPa) was measured for the sample containing 25% of the slag (S25). Presence of closed porosity, as a result of the incomplete bonding between the cement and the slag, that caused a substantial reduction of UCS was confirmed in the authors’ previous work [31,32]. Besides, it must be taken into account that in the waste S/S processes, the lowest required compressive resistance for the final S/S solids is 0.35 MPa [33]; all the tested samples met this value. The values of the UCS measured on these solidified products are close to the required strength of concrete of 15 MPa [34] in commercial and industrial structures. 

### 4.2. Leaching Test Results

#### 4.2.1. Toxicity Characteristic Leaching Procedure (TCLP)

The procedure, according to the TCLP [21] is applied as the standard test of solidified products (S10–S25). The concentrations of elements measured in the leachates are listed in Table 6. 

It was noticed that the concentrations of all the leached elements increased with the increase of the slag content in the solidified products. The major elements in the solutions were S (up to 11,671.6 mg L^−1^) and Na (up to 1865.4 mg L^−1^) ions which originated from the slag in the form of Na_2_SO_4_ [18]. High concentrations of Ca (up to 779.9 mg L^−1^) originate from the dissolution of the cement matrix, first of all, portlandite due to the negative ΔG_0_ values of the reaction of neutralization (Table 3) that indicated spontaneity of the dissolution process at standard conditions. The concentration of the toxic elements, Pb (in range 0.12 to 0.17 mg L^−1^) and As (in range 0.130 to 0.22 mg L^−1^), were less than S, Na, and Ca, and high below the limit values (5 mg L^−1^) (Table 6). According to these results, the effectiveness of the S/S treatment of the lead slag from the secondary lead production in cement-based materials was confirmed.

#### 4.2.2. Leaching under the Environmental Conditions Test

The leachability of the solidified product with the highest slag content (S25) was analyzed under environmental conditions in order to determine the actual leaching level of the elements in the real environment. The results of the LEC test after six months and one year are shown in Table 7. 

The concentrations of the selected elements in leachates obtained by the LEC test (Table 7) were not significantly changed from six months up to 12 months. The concentrations of S (5624 mg L^−1^ and 5462 mg L^−1^) were the highest. However, these values were lower than the values measured in solutions obtained by the TCLP test (Table 6). The same phenomenon was observed in the change of the Ca concentration, which was decreased in the leachates from the LEC test to 5.61 and 5.44 mg L^−1^. However, the concentrations of toxic elements, Pb (3.11 mg L^−1^) and As (8.91 mg L^−1^), were significantly higher in the solutions obtained under environmental conditions than in solutions obtained by the TCLP test (Table 6). At the same time, the pH values were increased up to a range of 12–13 (Table 7). The change in pH was identified as the main factor for the change of the toxic elements (Pb, As) leachability under the TCLP and LEC tests.

## 5. Discussion

### 5.1. Eh-pH Analyses 

**Figure 2:**
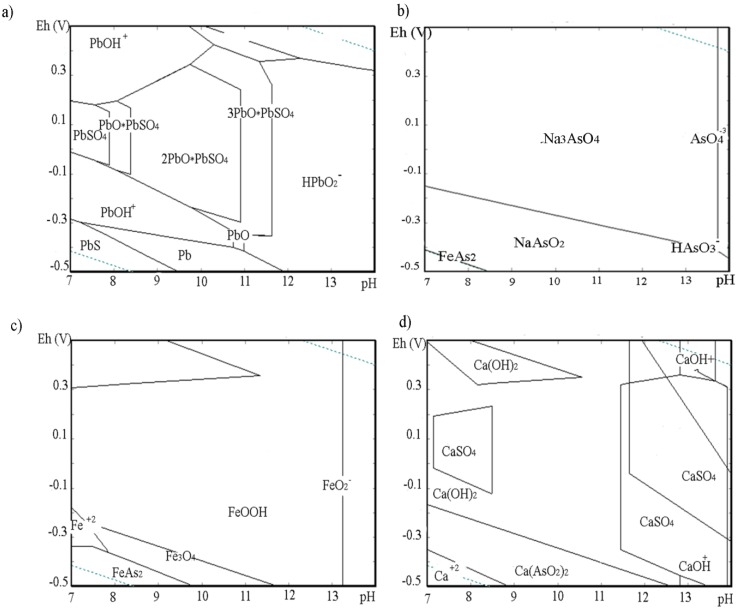
Eh-pH diagrams of (**a**) Pb, (**b**) As, (**c**) Fe, and (**d**) Ca species in the system As-Pb-S-Fe-Na-H_2_O at a temperature of 25 °C and a total pressure of 101.3 kPa, obtained by the TCLP and LEC tests.

Eh-pH analyses were based on the pH and the potential redox effect of the solubility of metal compounds (Pb, As, Fe and Ca) and their extraction capacity from the solidification product matrix. The Eh-pH diagrams of Pb, As, Fe, and Ca species in the system As-Pb-S-Fe-Na-Ca-H_2_O at a temperature of 25 °C and a total pressure of 101.3 kPa are shown in Figure 2. 

The pH of leachates obtained by the TCLP slowly increased in the range 8–9 with the addition of the slag. The redox potential was in the range from −15.51 to −17.58 mV. On the other side, the pH of the leachates obtained by the LEC test slowly decreased in the range of 12–13 with the leaching time (up to 12 months). That was followed with the Eh increasing from −314.4 to −278.3 mV.

Eh-pH analyses showed that the lead was stable in the form of the PbO·PbSO_4_ compound and arsenic in the form of sodium salt Na_3_AsO_4_, oxidation states As(V), at a pH range of 8–9 and Eh from −15.51 to −17.58 mV (Figure 2a,b). It was indicated that the potential precipitation process of these phases from solutions controlled the Pb and As solubility of the solidified products under determined conditions. 

The adsorption phenomena with the surface complexation model of Pb and As on Hfo and Surf [27,28,29,30] could be a reason for the low concentration of ions in the solutions (Table 6) due to favorable conditions in forming the FeOOH phase (Figure 2c). The base for Hfo formation was the iron from the slag (Table 2) [9,18]. It was noticed that the lead had small hydrated ions with high affinity towards physical adsorption, ion exchange, surface complexation, and precipitation [13]. Also, arsenate adsorption was related to the iron content of adsorbents and fitted by the Langmuir model by suggesting monolayer adsorption of arsenic onto adsorbents [35]. A more stable form of Ca ions was CaSO_4_ phase (Figure 2d).

**Figure 3:**
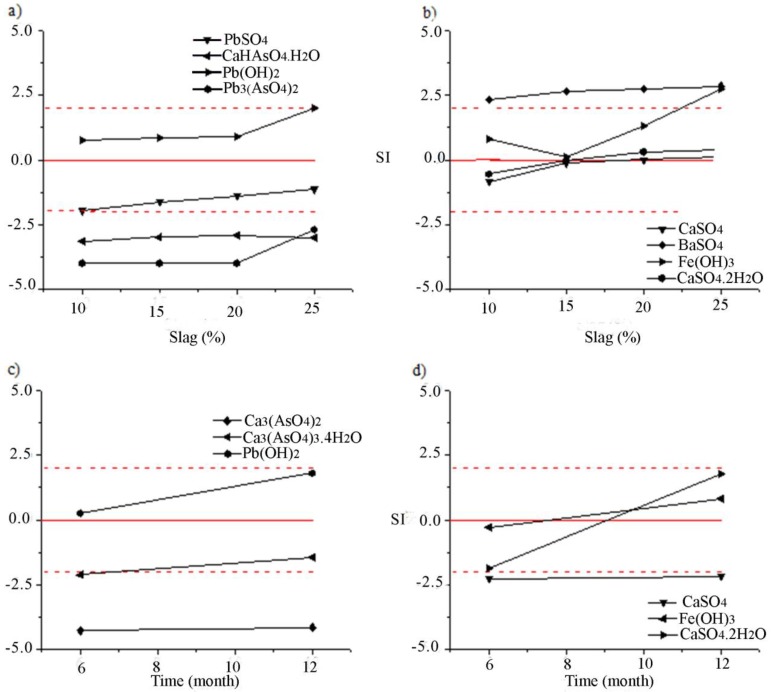
Saturation indices of minerals: (**a**) PbSO_4_, CaHAsO_4_·H_2_O, Pb(OH)_2_, Pb_3_(AsO_4_)_2_ and (**b**) CaSO_4_, BaSO_4_, Fe(OH)_3_, CaSO_4_·2H_2_O as a function of slag content into solidified samples (TCLP test); (**c**) Ca_3_(AsO_4_)_2_, Ca_3_(AsO_4_)_3_·4H_2_O, Pb(OH)_2_ and (**d**) CaSO_4_, Fe(OH)_3_, CaSO_4_·2H_2_O as a function of time (LEC test).

The effects of pH (12 to 13) and Eh (−314.4 to −278.3mV) on the speciation of Pb, As, Fe, and Ca species was also determined through Eh-pH diagrams (Figure 3). The pH values increased under environmental conditions and had a significant effect on the leachability of Pb due to its amphoteric nature [16] and forming its soluble species HPbO_2_^−^ (Figure 3a). Higher concentrations of As in solutions was a consequence of ion HAsO_3_^−^ stability (Figure 3b). In this range of Eh-pH, FeOOH was still stable and potentially available for adsorption of metal ions (Figure 3c). However, its adsorption power decreased with increasing pH values (above 13). Calcium ion remained stable in alkaline solutions in the form of CaSO_4_ (Figure 3d). The same effects of pH values on Pb and As release, with low solubility of Pb and As in the ranges of pH 8–10 and pH 3–7.5, respectively, and with higher mobility in acid and alkaline conditions, were observed in studies of De’an Pana et al. [5] and Sara Bisone et al. [30]. 

### 5.2. Modeling of Pb and As Leaching from Solidified Products (PHREEQC Program)

In this paper, the PHREEQC program was used to simulate the leaching of As and Pb from solidified products containing secondary lead alkaline slag in water. Cheryl E. Halim et al. [23] have developed a similar leaching model using the PHREEQC geochemical package to simulate the leaching of Pb, Cd, As, and Cr from cementitious wastes in the presence of both simple (0.1 and 0.6 M acetic acid) and complex municipal landfill leachates. In that model, both kinetic and thermodynamic parameters of key compounds, which were used in the subject research, were utilized and provided information on the leachate and precipitate species. 

PHREEQC simulations and thermodynamic data were used to determine the degree of saturation of leachates obtained by the TCLP (Table 6) and LEC (Table 7) tests. The simulation was based on the analysis of the phase’s precipitation capabilities, which may play an important role in the metals leaching behavior from the solidified product. Saturation indices (SI) of selected solubility-controlling phases were calculated by the PHREEQC program and shown in Table 8. Saturation indices of the minerals that are most likely to control for the constituent element solubility in solutions obtained by the TCLP and LEC tests are shown in Figure 3. 

The results of SI modeling for the TCLP test (Table 8, Figure 3a) indicated that the precipitation of phases (Fe(OH)_3_, FeOOH, Fe_2_O_3_, BaSO_4_, CaSO_4_, CaSO_4_·2H_2_O, and Pb(OH)_2_) with positive SI values was possible from solutions (pH 8.03 to 8.78; Eh −15.5 mV to −17.58 mV). In addition, forming of goethite (FeOOH) and CaSO_4_ was confirmed with Eh-pH analyses (Figure 2c,d). Modeling explained the low concentrations of Pb in leachates as a consequence of Pb(OH)_2_ precipitation. Potential precipitation of PbSO_4_ with SI values in the range −2.0 to 0.0 (Figure 3a) was confirmed with Eh-pH analyses (Figure 2a), as well as its adsorption on Hfo [16]. Saturation indices of As minerals (Table 8, Figure 3a) were not in the range from −2.0 to 2.0. The soluble forms of As minerals defined by modeling and low concentration As ions in solutions (Table 6) were in contradiction. Adsorption of As ions on Fe(OH)_3_ that precipitated (Figure 3a) and other forms of Hfo could explain this phenomenon. The most likely phase considered as the controlling mineral for As solubility was Pb_3_(AsO_4_)_2_ (Table 8, Figure 3a) due to the increase of its SI from −4.00 up to −2.70 with an increase in the content of slag in the solidified products. The concentration of S in the leachate determined the precipitation of CaSO_4_, CaSO_4_.2H_2_O, and BaSO_4_ from the solution.

Analyses of the leaching results obtained under environmental conditions (Table 8, for the LEC test; Figure 3b) showed that the following phases had positive SI values in solutions (pH range 12.06 to 12.80; Eh range −278.3 mV to −314.4 mV): FeOOH, Fe_2_O_3_, CaSO_4_·2H_2_O, and Pb(OH)_2_. In accordance with the modeling results, the precipitation of these phases controlled the concentrations of Ca, S, Fe, and Pb ions in the solution. Forming of goethite and CaSO4 was confirmed in Eh-pH analyses (Figure 2c,d). Higher concentrations of Pb ions in alkaline conditions were a consequence of its amphoteric nature [16] and the formation of the HPbO_2_^−^ ion in solutions at pH 12 (Figure 2a). The controlling minerals for As solubility were the hydrated forms of arsenates (Ca_3_(AsO_4_)_2_·4H_2_O) (Figure 3b) with SI −2.12 and 1.46, respectively (Table 8, LEC test). Also, adsorption of As ions on precipitated Fe(OH)_3_ (Figure 3b) and other forms of Hfo was possible. However, this phenomenon weakens with increasing pH values above 13 (Figure 2c).

**Figure 4:**
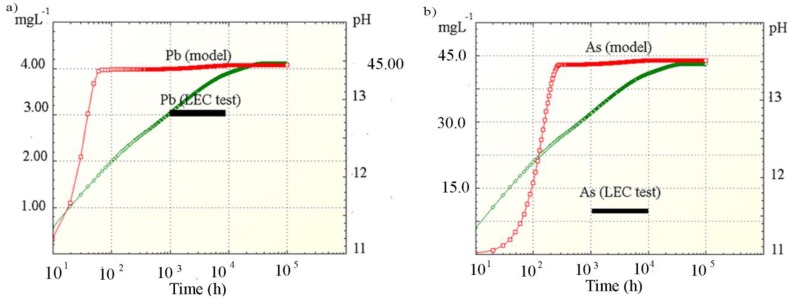
Simulated (**a**) Pb, and (**b**) As concentration profiles, pH values of leachates in the leaching process of the solidified productsS25 with distill water (PHREEQC program).

**Figure 5:**
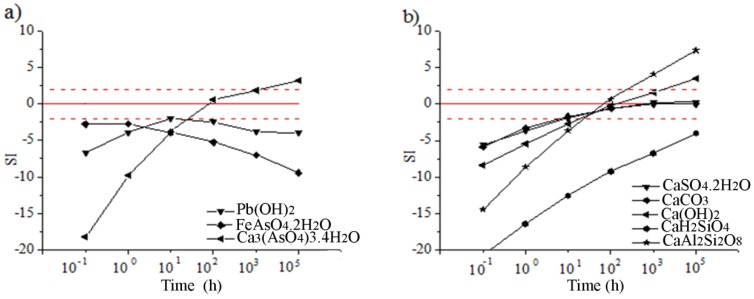
Saturation indices of minerals: (**a**) Pb and As, and (**b**) Ca as a function of leaching time (modeling results for S25).

The simulated Pb and As concentration profiles and pH values of the leachates in the leaching process of the solidified products S25 with distill water were determined by the equilibrium states of the possible equilibrium-controlling process in the PHREEQC program and displayed in Figure 4. Also, the concentrations of these elements leached under environmental conditions are shown in Figure 4. The dominant elements species in the solutions and the SI of selected compounds at different leaching stages are provided in Table 9 and Table 10. Saturation indices of minerals that are most likely to be controlling for constituent element solubility in the solutions obtained by the model are shown in Figure 5. 

The pH increase was simulated by the dissolution of the available portlandite, very early in the leaching process, with significant effects on the final pH solution. The thermodynamic parameters showed spontaneity of portlandite dissolution with an endothermic character (Table 3) due to negative ΔG_0_ values that indicated higher spontaneity of dissolution processes with temperature increase. With increasing time and pH, the calcite and CSH matrices also started to dissolve [23]. The model indicates that CSH dissolution kinetics is governed by OH^−^ concentration.

After 60 h of leaching, at pH 12, (Figure 4a), Pb release reached an equilibrium of 4.12 mg L^−1^. According to the model, Pb exists predominantly in solution as hydroxide complexes (Pb(OH)_3_^−^ and Pb(OH)_4_^−2^) (Table 9), due to its amorphous nature [13]. The negative SI values of Pb solid compounds (Table 10) indicate that the lead was soluble under this condition. Figure 5a shows that Pb(OH)_2_ was the mineral which could have the most impact on the solubility of this contaminant at high pH values with an SI around −3.00. Kovacevic et al. [25] and van Benschoten et al. [28] concluded that Pb adsorption on Hfo and Surf was one of the main controlling mechanisms of Pb release. However, this is not the case in this paper due to better matching of the modeling results with the concentrations of Pb leached under environmental conditions (Table 7, LEC test) when adsorption of Pb on Hfo and Surf was not included in the calculations. The low amounts of soluble Fe and Si in the system, available as adsorption sites, could be the reason for the mismatch with the results obtained when the adsorption was considered in the simulation process [30]. Also, at extremely high pH regions (pH > 13), the dissolution of metallic species embedded in different minerals controls the equilibrium conditions.

After 300 h of leaching, the simulated As concentration increased to 42.35 mg L^−1^ due to an elevated CSH dissolution rate at the high alkaline pH (up to 12.5), contributed by the “OH−” component in Equation (1).

The PHREEQC simulation suggests that As was released from the CSH matrix mainly as HAsO_4_^−2^ and CaAsO_4_− (Table 9). Furthermore, according to the model, the pH increase also resulted in the dissolution of scordite (FeAsO_4_·2H_2_O) precipitate and the formation of a Ca_3_(AsO_4_)_2_·H_2_O precipitate (SI above 0.0) (Figure 5). This is in accordance with conclusions of Saikia et al. [16] that the stability of the Ca_3_(AsO_4_)_2_·xH_2_O at high pH indicates the main role of Ca on the mobility of As. However, it can be seen (Table 9) that the SI’s of the Ca_3_(AsO_4_)_2_ remained well below 0.0 (Figure 5).

The simulation also indicated that the competition of the simultaneous precipitation of calcium as carbonate, hydroxide, sulfate and aluminosilicate (SI above 0.0) with calcium arsenate precipitation (Figure 5a,b) resulted in a high As concentration in the solution (up to 42.32 mg L^−1^) (Figure 4). Release of adsorbed As under high pH could also be the reason for high As concentration. 

The lower As concentration in solutions after the LEC test (8.91 and 8.63 mg L^−1^) (Table 7) compared with the results obtained by simulation (Table 7, LEC test) (Figure 4b) could be explained by As re-coprecipitation with the newly formed cement components (CaAl_2_Si_2_O_8_, CaH_2_SiO_4_) (Figure 5b) [36]. Ettringite and calcium silicate hydrate are the cement-based minerals that precipitated as secondary minerals during the leaching process of high calcium and silica-containing products and could accommodate oxyanions of As [16]. 

Based on the results obtained in this study, it was concluded that the addition of slag in solidified products increased its alkalinity (Table 6) due to the high alkalinity of the used slag [8,18]. The high addition of slag into solidified products was followed by an increase of sulfur content which lead to the formation of ettringite (Ca_6_[Al(OH)_6_]_2_(SO_4_)_3_·26H_2_O) into the solidified products [37]. Potential formation of ettringite can affect the decrease of mechanical properties and increase of toxic elements released from the solidified products. Also, the presence of SO_4_^−2^ ions in the leachate could influence the precipitation or sorption of oxyanions As on mineral surfaces with competitive effects [16]. In accordance with these observations, pre-treatment of the slag based on removing of its soluble phases (sodium sulfate salts) and soluble toxic species (especially sodium arsenates) before the use of slag in S/S processes for the preparation of concrete is the subject of further research by the authors.

## 6. Conclusions

This study was based on the assessment of the environmental impact of the use of alkaline slag from secondary lead production as a partial replacement of fine aggregates in cement-based stabilization/solidification (S/S) processes for the preparation of concrete.

The results of the compressive strength and leachability of toxic elements from the solidified products with up to 25% of slag, obtained by the standard TCLP test, indicated that the alkaline slag can be effectively stabilized and potentially used as a partial replacement of fine aggregates in cement-based constructions materials.

As efficient S/S treatment of the slag in cement-based products confirmed by laboratory research is not recommended for the safe use of lead slag construction materials in real-life, solidified products that contained 25% of the slag were leached under environmental conditions for one year. Increased leachability of pollutants from the solidified products was observed in the real environment (LEC) at high alkaline conditions (above 12) compared to its leachability assessed by the laboratory test (TCLP) at low alkaline conditions (around 8). The release of As from solidified products restricts the use of alkaline lead slag as an aggregate in concrete production.

Significant effects of pH on leachability of metal ions was confirmed by Eh-pH analyses and modeling results (PHREEQC program). The dominant mechanisms of metal immobilization at pH 8–9 were precipitation of Pb in forms of hydroxide and sulfates as well as As precipitation in the form of Na_3_AsO_4_, and its adsorption on the Hfo/Surf. The release of ions from the adsorbed surface, simultaneous precipitation of Ca_3_(AsO_4_)_2_·4H_2_O with other Ca phases (sulfates, carbonates, hydroxides), followed by the re-coprecipitation of As with cement phases were determined as phenomena with dominant effects on metals leachability at high pH (above 12).

The application of the alkaline slag in cement concrete or its long-term safe disposal that includes the slag pre-treatment based on removing of its soluble phases (sodium sulfate salts) and high content of soluble toxic species (especially sodium arsenates) before the S/S process is the subject of further research by the authors.

## Figures and Tables

**Table 1:** The corresponding proportion of fine aggregate and slag (in weight %) in solidified products.

Sample	Fine Aggregate	Slag
S0	40	-
S10	30	10
S15	25	15
S20	20	20
S25	15	25

**Table 2:** Composition of the slag and solidified samples (in weight %).

Element	Slag	S0	S10	S15	S20	S25
Pb	7.57 ± 0.46 ^a^	u.d.l.	0.76 ± 0.60	1.14 ± 0.47	1.52 ± 0.64	1.89 ± 0.47
As	0.10 ± 0.01	u.d.l.	0.01 ± 0.08	0.01 ± 0.01	0.02 ± 0.01	0.02 ± 0.01
Cr	0.10 ± 0.04	u.d.l.	0.01 ± 0.05	0.01 ± 0.34	0.02 ± 0.01	0.02 ± 0.01
Al	n.d.	3.61 ± 0.54	3.54 ± 0.55	3.42 ± 0.74	3.30 ± 0.39	3.18 ± 0.49
TIC	1.67 ± 0.19	u.d.l.	0.17 ± 0.35	0.25 ± 0.62	0.33 ± 0.41	0.45 ± 0.09
Ca	1.28 ± 0.54	47.80 ± 2.04	40.99 ± 1.32	38.56 ± 1.74	36.19 ± 1.37	33.79 ± 2.14
Fe	22.64 ± 0.99	3.96 ± 01.56	6.21 ± 0.54	7.18 ± 1.05	8.16 ± 0.97	9.13 ± 1.57
K	n.d.	0.17 ± 0.01	0.16 ± 0.17	0.16 ± 0.04	0.16 ± 0.03	0.16 ± 0.07
Mg	n.d.	1.18 ± 0.36	1.18 ± 0.51	1.14 ± 0.39	1.11 ± 0.81	1.07 ± 0.94
Na	23.81 ± 1.35	1.01 ± 0.87	3.38 ± 0.61	4.54 ± 0.34	5.71 ± 0.19	6.87 ± 1.02
Si	1.42 ± 0.51	7.46 ± 1.47	7.58 ± 1.40	7.25 ± 1.07	6.93 ± 1.46	6.60 ± 0.47
TS	10.95 ± 0.78	0.35 ± 0.04	1.44 ± 0.29	1.93 ± 0.18	2.42 ± 0.43	2.91 ± 0.67

^a^ Mean value and standard deviation *n* = 3; n.d.—not detected; u.d.l.—under detection limit.

**Table 3:** Initial mass of minerals (25% of solidified product) and thermodynamic data of reactions [22].

Matrices	In. Mass, g g^−1^_(solid. prod.)_	Reaction	log Kn or log Ksp	ΔG^0^, kJ mol^−1^	ΔH^0^_,_ kJ mol^−1^	ΔS^0^_,_ J K^−1^ mol^−1^
Ca(OH)_2_	149.67	Ca(OH)_2_ +2H^+^ = Ca^+2^ +2H_2_O	22.50 ^1)^	−128.84	−128.84	0.016
CaCO_3_	3.06	CaCO_3_ + H^+^ = Ca^+2^ + HCO_3_^−^	2.03 ^1)^	−11.58	−26.42	−49.74
C–S–H matrix						
CaH_2_SiO_4_	519.11	CaH_2_SiO_4_ + H^+^ = Ca^+2^ + H_3_SiO_4_^−^	15.30 ^1)^	−	−	−
Ca_3_Al_2_(H_4_O_4_)_3_	2.34	-	81.45 ^1)^	−	−	−
Ca_4_Al_2_Fe_2_O_10_	465.79	-	140.51^1)^	−	−	−
CaSO_4_·2H_2_O	132.68	CaSO_4_·2H_2_O = Ca^+2^ +SO_4_^−2^ +2H_2_O	−4.50 ^2)^	25.67	−1.76	−91.85
Mg(OH)_2_	39.83	Mg(OH)_2_ +2H^+^ = Mg^+2^ + 2H_2_O	16.83 ^1)^	−96.04	−113.99	−60.24
NaOH	169.95	NaOH = Na^+^ + OH^−^	6.93 ^2)^	−39.53	−44.52	−16.73
Pb(OH)_2_	0.04	Pb(OH)_2_ +2H^+^ = Pb^+2^ + 2H_2_O	13.28 ^1)^	−75.77	−54.75	70.53
Ca_3_(AsO_4_)_2_	0.92	Ca_3_(AsO_4_)_2_ + 4H^+^ = 3Ca^+2^ +2HAsO_4_^−2^	−18.947 ^1)^	108.138	−106.818	−720.965

^1)^ log neutralization constant (Kn); ^2)^ log solubility constant (Ksp).

**Table 4:** Dissolution rate constants of the matrix in the solidified product [23].

Matrices	A (m^2^g^−1^)	V (Lg^−1^)	Rate Constants (mol cm^−2^ s^−1^ M^−1^)				
			c_1_	c_2_	c_3_	c_4_	c_5_
CaCO_3_	400	10^−3^	10^−4.05^	10^−7.30^		10^−1.72^	10^−10.2^
Ca(OH)_2_	1300	10^−3^	10^−2.95^	-	-	-	10^−9.19^
C–S–H matrix	700000	10^−3^	10^−7.88^	-	10^–9.53^	-	-

**Table 5:** Thermodynamic data for the surface complexation constants for Hfo and Surf [27,28,29].

Adsorbed Species	Kf or Kd	Reaction
Hfo sOPb^+^	4.65 ^1)^	Hfo_sOP+ = Pb^+2^ + Hfo_sO^−^
Hfo wOPb^+^	0.30 ^1)^	Hfo_wOPb^+^ = Pb^+2^ + Hfo_wO^−^
Surf sOPb^+^	−7.75 ^1)^	Surf_sOPb^+^=Pb^+2^ + Surf_sO^−^
(Surf sO)_2_Pb	−17.23 ^1)^	(Surf_sO)_2_Pb =Pb+2 + 2Surf_sO^−^
Hfo wH_2_AsO_4_^−^	29.31 ^2)^	H_2_AsO_4_^−^ +Hfo_wOH= Hfo_wH_2_AsO_4_ +H_2_O
Hfo wHAsO_4_	23.51 ^2)^	H_3_AsO_4_ + Hfo_wOH = Hfo_wH_2_AsO_4_ + H_2_O
Hfo wOHAsO_4_	10.58 ^2)^	H_3_AsO_4_ + Hfo_wOH = Hfo_wOHAsO_4_^−3^ + 3H^+^

^1)^ log dissociation constant (Kd); ^2)^ log formation constant (Kf).

**Table 6:** The concentration of elements (in mg L^−1^) in the leachates obtained according to the TCLP test.

Element	Limit Value, mg L^−1^	Solidified Products			
		S10	S15	S20	S25
As	5.00	0.13	0.13	0.18	0.22
Ba	100	0.41	0.48	0.57	0.65
Ca	n.d. ^1)^	283.6	631.3	774.2	779.9
Fe	n.d. ^1)^	0.10	0.28	0.24	0.31
Na	n.d. ^1)^	489.4	1060.3	1188.3	1865.4
Pb	5.00	0.12	0.12	0.13	0.17
S	n.d. ^1)^	604.5	4637.1	5290.4	11671.6
Zn	250	0.12	0.11	0.13	0.71
pH		8.03	8.15	8.20	8.78
Eh, mV		−15.5	−16.02	−17.76	−17.58

^1)^ not defined.

**Table 7:** The concentration of elements (in mg L^−1^) in the leachates obtained according to LEC test.

Element	6 Months	12 Months
As	8.91	8.63
Fe	0.68	1.52
Pb	3.11	2.91
Zn	0.65	0.95
Ca	5.61	5.44
Ni	0.94	0.95
S	5624	5962
pH	12.80	12.06
Eh, mV	−314.4	−278.3

**Table 8:** SI’s of selected solubility-controlling phases under TCLP and LEC tests.

Phases	Formula	SI					
		TCLP of Solidified Products				LEC after Months	
		S10	S15	S20	S25	6	12
Anglesite	PbSO_4_	−1.97	−1.63	−1.40	−1.14	−10.73	−7.64
Anhydrite	CaSO_4_	−0.85	−0.12	0.01	0.11	−2.27	−2.18
Barite	BaSO_4_	2.34	2.64	2.75	2.86	n.d.	n.d.
Ca_3_(AsO_4_)	Ca_3_(AsO_4_)_2_	−5.59	−5.04	−4.76	−3.91	−4.29	−4.16
Ca_3_(AsO_4_)_2_·4H_2_O	Ca_3_(AsO_4_)_2_·4H_2_O	n.d.	n.d.	n.d.	n.d.	−2.12	−1.46
CaHAsO_4_·H_2_O	CaHAsO_4_·H_2_O	−3.14	−3.00	−2.94	−3.01	−6.08	−5.28
Fe(OH)_3_(a)	Fe(OH)_3_	0.80	0.12	1.30	2.76	−0.32	0.79
Goethite	FeOOH	6.69	6.87	7.19	8.66	5.20	6.32
Gypsum	CaSO_4_·2H_2_O	−0.55	−0.04	0.31	0.41	−1.85	1.77
Hematite	Fe_2_O_3_	15.39	15.97	16.38	19.32	12.36	14.58
Pb(OH)_2_	Pb(OH)_2_	0.76	0.84	0.90	2.04	0.26	1.80
Pb_3_(AsO_4_)_2_	Pb_3_(AsO_4_)_2_	−4.00	−4.00	−4.00	−2.70	−25.19	−16.04

n.d. not defined.

**Table 9:** Composition of solution and distribution of species vs. the leaching stages (modeling results).

Time, h	10^−1^	10^0^	10^1^	10^2^	10^3^	10^5^
pH	9.41	10.37	11.33	12.19	12.82	13.46
pe	8.88	7.93	6.81	5.95	5.58	5.30
Dominant species	molality					
As(3)	9.86 × 10^−37^	3.97 × 10^−36^	6.68 × 10^−36^	5.63 × 10^−36^	6.43 × 10^−37^	4.93 × 10^−40^
H_2_AsO_3_^−^	5.99 × 10^−37^	3.64 × 10^−36^	5.42 × 10^−36^			
HAsO_3_^−2^				3.50 × 10^−36^		
AsO_3_^−^					3.42 × 10^−37^	4.93 × 10^−40^
As(5)	3.94 × 10^−10^	3.53 × 10^−8^	3.22 × 10^−6^	2.14 × 10^−4^	5.74 × 10^−4^	5.85 × 10^−4^
HAsO_4_^−2^	3.85 × 10^−10^	2.46 × 10^−8^				
CaAsO_4_^−^			2.79 × 10^−6^	2.07 × 10^−4^	5.62 × 10^−4^	5.85 × 10^−4^
Pb	1.97 × 10^−10^	1.76 × 10^−8^	1.56 × 10^−6^	1.98 × 10^−5^	1.99 × 10^−5^	2.03 × 10^−5^
Pb(OH)^+^	1.90 × 10^−10^	1.57 × 10^−8^				
Pb(OH)_3_^−^			8.67 × 10^−7^			
Pb(OH)_4_^−2^				1.72 × 10^−5^	1.94 × 10^−5^	1.94 × 10^−5^

**Table 10:** SI’s of selected compounds at different stages of leaching time (modeling results).

Phase	Formula	10^−1^ h	10^0^ h	10^1^ h	10^2^ h	10^3^ h	10^5^ h
Calcite	CaCO_3_	−5.87	−3.32	−1.69	−0.62	−0.01	0.00
Portlandite	Ca(OH)_2_	−8.35	−5.47	−2.68	−0.25	1.52	3.59
Anhydrite	CaSO_4_	−5.88	−3.97	−2.25	−0.91	−0.09	0.02
Gypsum	CaSO_4_·2H_2_O	−5.57	−3.66	−1.95	−0.61	0.21	0.27
Anorthite	CaAl_2_Si_2_O_8_	−14.43	−8.63	−3.68	0.68	4.06	7.34
Ca_4_Al_2_Fe_2_O_10_	Ca_4_Al_2_Fe_2_O_10_	−53.37	−41.46	−30.19	−20.21	−12.36	−4.09
CaH_2_SiO_4_	CaH_2_SiO_4_	−20.77	−16.46	−12.60	−9.26	−6.77	−4.04
Ca_3_(AsO_4_)_2_	Ca_3_(AsO_4_)_2_	−31.73	−23.39	−17.32	−12.97	−11.68	−10.24
Ca_3_(AsO_4_)_2_·4H_2_O	Ca_3_(AsO_4_)_2_·4H_2_O	−18.20	−9.86	−3.79	0.56	1.84	3.19
CaHAsO_4_·H_2_O	CaHAsO_4_·H_2_O	−9.28	−6.55	−4.91	−3.95	−4.19	−4.55
Scordite	FeAsO_4_·2H_2_O	−2.79	−2.76	−3.90	−5.27	−7.01	−9.47
Gibbsite	Al(OH)_3_	0.23	0.26	0.27	0.32	0.40	0.30
Fe(OH)_3_	Fe(OH)_3_	1.16	1.32	1.36	1.45	1.73	1.70
Goethite	FeOOH	7.82	7.98	8.02	8.11	8.39	8.39
Hematite	Fe_2_O_3_	17.65	17.97	18.04	18.23	18.08	18.81
Cerrusite	PbCO_3_	−9.51	−6.98	−6.30	−8.08	−10.63	−12.79
Anglesite	PbSO_4_	−10.66	−8.76	−7.99	−9.52	−11.84	−13.91
Pb(OH)_2_	Pb(OH)_2_	−6.73	−3.86	−2.01	−2.45	−3.83	−3.93
Pb_3_(AsO_4_)_2_	Pb_3_(AsO_4_)_2_	−26.58	−18.28	−15.04	−19.28	−27.45	−32.53
Pb_3_(CO_3_)_2_(OH)_2_	Pb_3_(CO_3_)_2_(OH)_2_	−48.56	−40.62	−37.41	−41.41	−47.89	−52.31
PbCO_3_	PbCO_3_	−9.11	−6.57	−5.89	−7.67	−10.22	−12.38
PbSiO_3_	PbSiO_3_	−11.92	−11.66	−11.89	−12.18	−12.54	−122.20
